# Social Determinants of ART Adherence among Black Adults with HIV

**DOI:** 10.21203/rs.3.rs-6591266/v1

**Published:** 2025-06-06

**Authors:** Vanessa B. Serrano, Maximo Prescott, Dafna Paltin, Astrid Maves, Carlos Rivera-Saldana, Samantha Yeager, Jessica L. Montoya, David J. Moore

**Affiliations:** San Diego State University/University of California San Diego Joint Doctoral Program in Clinical Psychology; San Diego State University/University of California San Diego Joint Doctoral Program in Clinical Psychology; San Diego State University/University of California San Diego Joint Doctoral Program in Clinical Psychology; University of California, San Diego; University of California, San Diego; University of California, San Diego; University of California, San Diego; University of California, San Diego

**Keywords:** HIV, Black/African American, Social Determinants of Health

## Abstract

**Background:**

Black people with HIV (PWH) show poorer rates of antiretroviral therapy (ART) adherence compared to other racial/ethnic groups in the United States. Social determinants of health (SDOH) may influence ART adherence, and disparities in SDOH are known to disproportionately impact Black communities. The iC-CHANGE study evaluated the efficacy of a personalized, culturally adapted, text messaging intervention to improve ART adherence among Black PWH and incorporated self-report measures of ART adherence at study visits. This analysis sought to examine the relationship between SDOH and longitudinal ART adherence measures.

**Methods:**

Participants (90 Black PWH) were sent text messages through a two-way, fully automated text-messaging intervention, and attended in person study visits (12-week intervals). Measures of ART adherence included a visual analog scale, 3-item quantitative questionnaire, and text message responses. The relationship between SDOH and ART adherence measures were evaluated using linear and logistic mixed effects regression models.

**Results:**

Majority of the participants (n=90) were male (82.0%) and mean (SD) age was 46.5 (11.7) years at baseline. Housing stability was significantly associated with ART adherence, such that individuals without stable housing had lower scores on the 3-item self-report adherence scale (β = −4.04, *p* = .001), the visual analog adherence scale (β = −4.15, *p*= .02), and text message responses (β = −.03, *p* =.02) compared to individuals with stable housing. Older age was also associated with higher adherence on the visual analog scale (β = 0.003, p = .008), but lower adherence in daily text message responses (β = −0.06, p = .03). Health literacy also showed a positive association with ART adherence as measures by text message responses (β = 0.03, p < .02).

**Conclusion:**

These findings highlight the need for targeted interventions addressing housing stability and health literacy to improve ART adherence among Black individuals with HIV. Integrating these factors with clinical care may significantly enhance treatment outcomes and contribute to health equity.

## Introduction

African American/Black (henceforward referred to as Black) people are disproportionately affected by HIV in the United States. In 2022, Black individuals made up 14% of the U.S. population yet accounted for almost 40% of new HIV diagnoses and people with HIV (1). Black people with HIV (PWH) also experience increased risks for suboptimal HIV-related outcomes, including challenges with antiretroviral treatment (ART) adherence. A smaller percentage of Black American PWH achieve viral suppression compared to their Hispanic/Latinx or White counterparts. In 2022, only 53% of Black PWH were virally suppressed, compared to 63% of White PWH (2). One estimate indicated that Black PWH had 1.61 times the odds of suboptimal (< 95%) ART adherence compared to White PWH (3).

Social determinants of health (SDOH) are the nonmedical factors in the environments where people are born, live, learn, work, interact, and age that influence health outcomes (4). SDOH can be grouped into five domains: economic stability, education, healthcare access and quality, neighborhood and built environment, and social/community support. Black PWH frequently experience suboptimal SDOH in each of these domains, driving health disparities (5). For example, HIV-related outcomes have been tied to SDOH such as access to healthcare, affordable housing, stigmatizing attitudes towards HIV, social support, community cohesion, and self-efficacy for managing health (6, 7). For Black PWH, suboptimal SDOH have been associated with lower receipt of, and poorer adherence to, ART (8, 9). Various SDOH measures exist, with differences in the specific variables used to capture SDOH domains (10). Specific to PWH, some groups have selected HIV-specific variables as potential SDOH indicators (e.g., HIV-related discrimination being used as a measure of community support; (5). Using similar measures, understanding the influence of SDOH on ART adherence is critical to better support the HIV outcomes of Black adults in HIV care.

Methods for supporting and measuring ART adherence are limited, especially for mobile health (mHealth) interventions. Current interventions using text messaging to improve ART adherence target various demographics of PWH. Most interventions show improved adherence, with text message reminders proving to be an effective form of support (11). However, many studies lacked comprehensive adherence results or demographic-specific analyses. Individual-level factors such as race, age, and substance use behaviors have emerged as significant predictors of adherence and program engagement. Overall, personalized and frequent text messaging appears to be a promising strategy to enhance ART adherence among PWH. However, further research is needed to explore demographic nuances and specifically SDOH as predictors of intervention success to optimize the effectiveness of these interventions. This project builds on current literature by examining the efficacy of an mHealth intervention to improve ART adherence that was culturally tailored for Black PWH.

The Individual Community Care for HIV/AIDS Now: Getting Engaged (iC-CHANGE) study recruited a longitudinal cohort of Black PWH to participate in a mHealth study to promote ART adherence. The individualized Texting for Adherence Building (iTAB) intervention was developed as a two-way short message service (SMS) text messaging intervention to improve ART adherence and associated virologic suppression among PWH (12–15). To address lower levels of engagement in HIV care among Black PWH in Southern California, iTAB was culturally adapted for Black PWH in the iC-CHANGE study (15). Prior analyses of the iC-CHANGE data suggested various psychosocial determinants of health (i.e., self-efficacy, medical mistrust) were associated with ART adherence among Black PWH (16). Despite informative findings from iC-CHANGE and existing research, critical gaps in understanding modifiable determinants of ART adherence among Black PWH exist.

Considering the potential influence of SDOH on HIV care outcomes, particularly among Black PWH, we sought to examine the role of SDOH in ART adherence examining three self-report measures of ART adherence across the longitudinal iC-CHANGE study as the outcomes (i.e., a 3-item self-report measure, a visual analog scale (VAS), and daily text message responses to the mobile phone intervention). Guided by existing measures, we modeled each SDOH domain: education (as measured by health literacy and years of education), economic stability (as measured by family income), healthcare access (as measured by healthcare utilization), neighborhood/built environment (as measured by housing stability), and community support specific to HIV (as measured by HIV disclosure). Considering previous findings of iC-CHANGE, it was hypothesized that SDOH specific to healthcare and social/community context would be the strongest predictors of ART adherence.

## Methods

### Participants

Participants were 90 Black/African American adults with HIV who were actively receiving care for HIV from November 2017 to July 2019 at Family Health Centers San Diego (FHCSD) in San Diego, CA, a community health center focused on aiding low-income and medically underserved communities. Recruitment for the iC-CHANGE text messaging intervention was completed through patient pools of Black/African American PWH at FHCSD. All participants were required to have been taking ART for at least two months at the time of enrollment. Participants were required to be English speaking and able to provide informed consent. Those with obstacles to their ability to participate in all visits such as severe neurocognitive impairments like dementia were excluded from enrollment. Similarly, those who were not be able to complete the entire duration of the study or would not be present in San Diego for the necessary 24 or 48 weeks were excluded as well. All participants provided written informed consent to participate, and were treated in accordance with the American Psychological Association’s Ethical Principles of Psychologists and Code of Conduct. All study procedures received Institutional Review Board approval by University of California San Diego (IRB #161608).

### Measures

#### Social Determinants of Health.

While previous studies have conceptualized and measured specific SDOH in various ways, our SDOH indicators were guided by existing literature on SDOH specific to PWH (5, 17). As such, we identified measures collected at baseline that corresponded to the five key domains of SDOH as hypothesized predictors of ART adherence:

#### Education.

Total number of years of education (collected in a standard demographic questionnaire; Supplementary File 1) and health literacy were examined to represent education. Health literacy was measured using the Short Assessment of Health Literacy-English (SAHL-E), an 18-item assessment intended to measure a participant’s understanding of medical terms (18). SAHL-E measures understanding of these medical terms by assessing for correct pronunciation and asking participants to select which out of the two provided words is most similar or has a closer association to a target medical term (e.g. if the target word is *syphilis* and the two provided words are *contraception* and *condom*, the correct answer would be *condom*). Correct answers receive a score of 1 while “*don’t know*” and wrong answers—like associating *syphilis* with *contraception*, or mispronouncing syphilis—would be counted as 0. SAHL-E scores are calculated by totaling the number of correct answers with the highest score possible being 18 and scores between 0 and 14 indicating low health literacy.

#### Economic Stability

Family income (Supplementary File 1) was used as a measure of economic stability, with demographic questionnaire options ranging from less than $10,000 to $150,000 or more.

#### Healthcare Utilization

While all participants were receiving care at FHCSD, variations in individuals’ level of healthcare utilization were captured using Utilizations of Care, a 9-item self-report questionnaire from the HIV Cost and Services Utilization Study (HCSUS; RAND, 1999). Items asked participants about their usage of services in the past 6 months such as dental care, alternative therapies (e.g. acupuncture), doctors’ visits, the emergency room, etc. Participants would answer “yes” or “no” to the usage of these services. Higher scores would indicate more healthcare utilization in the past 6 months.

#### Housing Stability

Housing stability was examined as a measure of neighborhood/built environment. Specifically, individuals shared their usual sleeping location [stable housing (e.g., in your own apartment, room, or house) versus less stable housing (e.g., in a friend or relative’s housing, in a hotel/motel room, in a car/vehicle)] in the standard demographic questionnaire (Supplementary File 1). Based on these responses, individuals were categorized as having stable housing or lacking stable housing.

#### Community Support (specific to PWH)

Community support was captured through the HIV disclosure subscale as measured by a questionnaire relating to HIV stigma (19). While not a direct measure of community support, HIV disclosure reflects an individual’s level of social engagement and openness about their HIV status, which can influence access to support systems and receipt of support relating to HIV. Participants were asked whether they had disclosed their HIV status to various individuals or groups, including people living with them, family members, friends, or colleagues at work. Based on their responses, participants were categorized as either having disclosed their HIV status or not disclosed it.

### Outcomes:

#### 3-item Adherence Scale.

ART adherence over the past 30 days was measured using a 3-item ART adherence scale (20, 21). This scale was administered at baseline and then every 12 weeks over the course of the study. The first item asked participants how many days in the past 30 they missed at least one dose of their HIV medication, with responses ranging from 0 to 30. The second item assessed how well they believed they followed their prescribed medication regimen, with Likert-type responses ranging from “very poor” to “excellent.” The third item asked participants how often they took their medication as prescribed, with responses ranging from “never” to “always.” Consistent with Wilson et al.’s scoring procedures (2020), responses to these three adherence items were transformed to a 0–100 scale, where 0 indicated the lowest adherence and 100 indicated perfect adherence. A summary adherence score was calculated as the average of the three individual items, and the adherence score was analyzed as a continuous variable. This longitudinal adherence score served as an outcome measure in our analyses.

#### Visual Analog Scale (VAS).

A visual analog scale (VAS; Supplementary File 2) provided another measure of participant self-reported adherence. The VAS was administered at baseline and then every 12 weeks over the course of the study. Participants would mark an “X” on a line with anchors from 0–100 indicating what percentage of their HIV medications they had taken in the past month. The “X” on the VAS would then correspond to what percent of their medication participants perceived themselves to have taken in the past month (i.e., placing an X on the middle of the line would be counted as “50%”). These longitudinal participant-plotted percentage scores served as an outcome measure in our analyses.

#### Text Messaging Adherence.

Text messaging through the iC-CHANGE iTAB messaging system was used to measure self-reported adherence to ART (12–15). Participants chose to participate in either the 24-week or 48-week text-messaging intervention with a majority choosing the 48-week duration. Participants would respond with “Y” (indicating yes), “N” (indicating no), or “S” (indicating snooze, i.e., a request to receive a text message in one hour) in response to text message prompts of whether they had taken their medication. No (“N”) responses would receive an encouraging medication adherence message prompting them to reach out to their care team (e.g. “*Please take a moment for your health ASAP. Call us if you need assistance or have a question*”). Three consecutive no (“N”) responses, or nonresponse, would trigger an automated message about study noncompliance. In addition to the message, FHCSD care coordinators would contact the participant about their noncompliance and discuss potential barriers to engagement. Participants were considered adherent to ART for a given day if they responded “Y” and non-adherent if they either did not respond or responded “N” via text message with iTAB. The proportion of “Y” responses over a 12-week interval was calculated, resulting in up to four “epochs” of ART adherence measures across the 48-week study. These longitudinal epochs served as an outcome measure in our analyses.

### Statistical Analysis

Mixed effects linear and logistic models were used to evaluate changes in self-reported adherence measures over study visit. The mixed effects models evaluated a linear time effect with random intercepts (i.e., random subject effect). Demographic variables were screened for their univariate association with the outcome (i.e., ART adherence) and were included in the models if the variable had a significant result (p > .2) in either analysis.

## Results

Participant demographics are reported in Table 1. Of covariates screened, only age at baseline was found to be significantly associated with the outcome (i.e., ART adherence as measured by the 3-item adherence self-report scale and the VAS), and thus included in the final models.

The first mixed effects model (SDOH predicting the 3-item self-report scale scores across study visits; Table 2), with a linear time effect and random intercepts indicated a significant main effect of housing instability (β = −4.04, *p* < .001). The main effect of housing stability suggests a significant difference in ART adherence as measured by the 3-item self-report scale across study visits, such that individuals who lacked consistent housing had lower adherence (i.e., a 4-point decrease in the self-report adherence measure) than individuals who identified having stable housing. No other significant main effects were observed.

For our second model (SDOH predicting VAS scores; Table 2), the mixed effects model with a linear time effect and random intercepts indicated a significant main effect of housing stability (β = −4.15, *p* = .02) and age (.003 *p* < .001). The main effect of housing stability suggests a significant difference in ART adherence as measured by the VAS across study visits, such that individuals who lacked consistent housing had lower adherence than individuals who reported having stable housing. Similarly, the main effect of age indicated that individuals who are older report greater ART adherence as measured by the VAS across study visits (β = 0.003, p = .008).

The third model (SDOH predicting daily text message responses; Table 2) used a mixed effects logistic regression model with a linear time effect and random intercepts. In this model, housing stability was statistically significant (β = − .03, *p* = .02), indicating that individuals who lacked consistent housing had a lower proportion of “yes” text message responses compared to those who were stably housed. Further, greater health literacy was associated with greater ART adherence as measured by text message responses (β = − .03, *p* < .001). Age also remained a significant predictor in this model, such that individuals of older ages reported lower ART adherence (β = −0.06, p = .03).

## Discussion

The current longitudinal study employed linear and logistic mixed effects regression models to examine the association between SDOH and ART adherence among Black PWH who participated in a daily text messaging intervention to support ART adherence. Our results reveal that housing stability is a critical factor influencing ART adherence, as demonstrated by the significant associations observed among a 3-item self-report measure of ART adherence, a VAS, as well as the text message response model. Other significant predictors of ART adherence included age and health literacy. Taken together, this research may help to identify specific SDOH that may be targeted for improvement to better support ART adherence among Black PWH.

Our finding that housing stability is a salient predictor of ART adherence aligns with prior research emphasizing the significant impact of housing instability on health outcomes and medication adherence. Most broadly, access to housing is a critical SDOH that can affect mental and physical health across individuals regardless of HIV status (22–24). Experiencing housing instability contributes to stress, anxiety, and other mental health symptoms that negatively impact physical health outcomes (24). Related, a systematic review and meta-analysis examining multiple chronic health conditions (inclusive of 9 studies specific to PWH) reported that alongside food insecurity, housing instability most consistently impacted medication adherence (25). Specific to PWH, Galarraga et al. (26) established that unstable housing is a significant predictor of HIV biomarkers (i.e., CD4 cell count, viral load). Related, housing instability has been reported to impact overall retention in HIV care (27). The Rajabiun et al. (2018) study found that not only was housing stability associated with significantly higher rates of ART prescriptions and viral suppression, but also retention in care (compared to individuals who remained or became unstably housed during the study period (27). Further explaining these similar findings, housing instability may complicate health disparities by creating environments that hinder access to health services, increasing the likelihood of missed appointments, and leading to inconsistent ART adherence (28, 29). This is particularly relevant for PWH, where consistent ART adherence is crucial for therapeutic efficacy and viral suppression. In further support of the strong influence of housing on ART adherence, a systematic review by Aidala et al. (30) corroborates these findings by demonstrating that lack of stable housing is a significant barrier to remaining in HIV care, ART adherence, and viral suppression, and risk of forward transmission. Studies highlighted in the Aidala review (30) expand that individuals experiencing housing instability face significant challenges such as increased stress, disrupted access to healthcare, and unstable living conditions, all of which can adversely affect their ability to adhere to prescribed treatments. The review’s findings underscore that individuals without stable housing frequently encounter difficulties in maintaining consistent healthcare practices, which aligns with our observation of lower ART adherence among those experiencing unstable housing. Taken together, our findings are supported by prior research suggesting the importance of housing on ART adherence, and further extends such research by specifically examining Black PWH—a population disproportionately impacted by HIV and health disparities.

Health literacy was significantly associated with ART adherence in our text message response models, and this finding aligns with existing research linking higher health literacy with better health outcomes and adherence to treatment among PWH (31, 32). For Black PWH, however, health literacy is often intertwined with broader socio-economic and systemic factors. Research shows that Black individuals face unique barriers to effective health literacy, including historical mistrust of the healthcare system, cultural differences in health communication, and disparities in access to quality health education (33). These factors may exacerbate challenges in navigating treatment regimens and healthcare systems. Across diverse health conditions among Black adults, culturally tailored and interactive approaches such as storytelling and culturally appropriate graphics can improve health outcomes by making health information more relevant and accessible to target populations (33). Such tailored interventions can help bridge gaps in understanding and enhance ART adherence among Black PWH. Thus, our research in combination with existing research supports the need for culturally competent health literacy programs that take into account the unique experiences and needs of Black communities, and specifically, Black PWH.

Age showed differential associations with ART adherence, such that older age was associated with higher adherence in the VAS model but lower adherence in the text message responses model of ART adherence. Previous research supports the notion that older adults often face unique challenges related to daily ART adherence, including managing complex medication regimens and comorbidities (34). Thus, the differential association of age with the study’s different adherence measures suggests that older adults might experience barriers in responding to daily, real-time adherence tracking methods like text messages, potentially due to technological barriers or cognitive decline associated with age (35), and instead have higher perceptions of adherence when asked to subjectively recall their adherence during study visits. Taken together, particularly older adults in this population may benefit from tailored attention to methods of supporting ART adherence depending on their familiarity and comfort with technology.

Despite the contributions of our research to the existing body of literature on SDOH among Black PWH, there are limitations to the present study. First, participants were already receiving care within FHCSD, and thus may not be representative of the broader population of Black PWH across the care continuum (e.g., individuals who have yet to be linked to consistent care). While this is a limitation, it is notable that participants self-selected to be a part of this study due to perceiving themselves to have difficulty with ART adherence. Second, relating to the 3-item self report measure of adherence as well as VAS, participants completed these measures every 12 weeks during their regular visits to FHCSD. As such, it is possible that recall biases potentially influenced their self-reports. Third, our strongest and most consistent indicator of ART adherence across models of ART adherence was housing stability, as measured by consistent sleeping location. While these findings are meaningful and informative for identifying SDOH that are most salient for ART adherence, little is known of the longitudinal histories of our participants and their housing stability (e.g., how long they had been unstably housed). Finally, while text messaging data was carefully monitored and participants were asked to provide feedback if they encountered any technical difficulties, given the nature of the automated text messaging system that generated text message responses, there is potential for occasional technical errors that prevented an accurate measure of ART adherence (e.g., if a text message was not sent as planned).

Our study underscores the critical importance of housing stability, age, and health literacy as key factors influencing ART adherence among Black PWH. Interventions that address suboptimal SDOH (e.g., housing stability) have the potential to improve or support ART adherence. For instance, programs aimed at improving housing stability could be integrated with health literacy initiatives. Such comprehensive approaches are crucial for overcoming the barriers that exacerbate health disparities and for fostering environments that facilitate rather than hinder ART adherence. Additional longitudinal studies are essential to gain a deeper understanding of how SDOH (e.g., housing stability) and individual-level factors (e.g., age) interact over time and to assess the long-term impact of comprehensive interventions on ART adherence. By addressing these SDOH in clinical care, we can work towards more effective strategies that not only improve adherence but also contribute to better overall health outcomes for Black PWH.

## Figures and Tables

**Table 1.** Participant characteristics

Variable	Descriptive Statistics (n = 90)
48-week study (vs. 24-week study)	83 (92%)
Age (median)	50 years (IQR: 36–57, range 23–66)
Gender identity	
Male	74 (82%)
Female	12 (15%)
Genderqueer/gender non-conforming	1 (1%)
Transwoman	1 (1%)
Education	
Completed high school/ GED	8 (8.9%)
Some college	18 (19.8%)
Completed college	47 (51.6%)
Some graduate education	10 (11%)
Graduate degree	2 (2.2%)
Ethnicity other than Black	
Hispanic/Latino/Spanish	9 (10%)
American Indian or Alaska Native	9 (10%)
Asian American or Asian Origin	1 (1%)
White	3 (3%)
Other	5 (5%)
Income	
<$10,000	41 (46%)
$10,000 to $19,999	26 (29%)
>$20,000	22 (24%)
Employment status	
Full-time employment	19 (20%)
On disability	26 (28%)
Unemployed	27 (29%)
Relationship status	
Single	69 (76%)
In a committed relationship	16 (17%)
Other	5 (5%)
HIV disclosure	
Ever disclosed	82 (90%)
Disclosed to family	66 (80%)
Disclosed to friends	72 (87%)

**Table 2:** Final model for each model of ART adherence.

	3-Item Self-report Scale		Visual Analog Scale		Text Message Responses	
	β	*p*	β	*p*	β	*p*
*Education*	−1.57	.21	.94	.43	− .01	.32
*Health Literacy*	.87	.51	1.29	.28	.03	**< .001**
*Economic Stability*	.99	.37	.56	.58	.02	.11
*Healthcare Utilization*	1.27	.13	.82	.46	.01	.53
*Housing Stability*	−4.04	**.001**	−4.15	**< .001**	− .03	**.02**
*Community Support*	−9.40	.21	−7.87	.26	.04	.58

Note: Age was significantly associated with both the visual analog scale and text message responses, and thus included in both final models as a covariate.

## Data Availability

The datasets used and/or analyzed during the current study are available from the corresponding author on reasonable request.
